# The Orphan Nuclear Receptor LRH-1 and ERα Activate GREB1 Expression to Induce Breast Cancer Cell Proliferation

**DOI:** 10.1371/journal.pone.0031593

**Published:** 2012-02-16

**Authors:** Ashwini L. Chand, Dhilushi D. Wijayakumara, Kevin C. Knower, Kerrie A. Herridge, Tamara L. Howard, Kyren A. Lazarus, Colin D. Clyne

**Affiliations:** Prince Henry's Institute, Monash Medical Centre, Clayton, Victoria, Australia; Emory University, United States of America

## Abstract

**Background:**

Liver Receptor Homolog 1 (LRH-1, NR5A2) is an orphan nuclear receptor that is over-expressed in cancers in tissues such as the breast, colon and pancreas. LRH-1 plays important roles in embryonic development, steroidogenesis and cholesterol homeostasis. In tumor cells, LRH-1 induces proliferation and cell cycle progression. High LRH-1 expression is demonstrated in breast cancers, positively correlating with ERα status and aromatase activity. LRH-1 dependent cellular mechanisms in breast cancer epithelial cells are poorly defined. Hence in the present study we investigated the actions of LRH-1 in estrogen receptor α (ERα) positive breast cancer cells.

**Results:**

The study aimed to investigate LRH-1 dependent mechanisms that promote breast cancer proliferation. We identified that LRH-1 regulated the expression of Growth Regulation by Estrogen in Breast Cancer 1 (GREB1) in MCF-7 and MDA-MB-231 cells. Over-expression of LRH-1 increased GREB1 mRNA levels while knockdown of LRH-1 reduced its expression. GREB1 is a well characterised ERα target gene, with three estrogen response elements (ERE) located on its promoter. Chromatin immunoprecipitation studies provided evidence of the co-localisation of LRH-1 and ERα at all three EREs. With electrophoretic mobility shift assays, we demonstrated direct binding of LRH-1 to EREs located on GREB1 and Trefoil Factor 1 (TFF1, pS2) promoters. LRH-1 and ERα co-operatively activated transcription of ERE luciferase reporter constructs suggesting an overlap in regulation of target genes in breast cancer cells. Over-expression of LRH-1 resulted in an increase in cell proliferation. This effect was more pronounced with estradiol treatment. In the presence of ICI 182,780, an ERα antagonist, LRH-1 still induced proliferation.

**Conclusions:**

We conclude that in ER-positive breast cancer cells, LRH-1 promotes cell proliferation by enhancing ERα mediated transcription of target genes such as GREB-1. Collectively these findings indicate the importance of LRH-1 in the progression of hormone-dependent breast cancer and implicate LRH-1 as a potential avenue for drug development.

## Introduction

Exposure of breast tissue to circulating hormones is a key risk factor in breast cancer incidence [1], [2], [3]. Therefore understanding the mechanisms of hormonal actions is critical in progress towards better treatment options. In this report we analysed the effect of the orphan nuclear receptor NR5A2 (also termed Liver Receptor Homolog-1, LRH-1) on the transcriptional regulation of Growth Regulation by Estrogen in Breast Cancer (GREB1) and breast cancer proliferation.

LRH-1 belongs to the NR5A subclass of nuclear receptors and regulates gene transcription by binding as a monomer to an extended nuclear receptor half-site, consensus YCAAGGYCR [4]. LRH-1 has well established roles in metabolic pathways involved in bile acid synthesis [5], [6] and reverse cholesterol transport [7], [8]. It is highly expressed in the ovary where it is vital for the regulation of steroidogenesis [9], [10]. In embryonic tissue it causes the differentiation of enterohepatic tissue [11], [12] and pluripotency in embryonic stem cells [13], [14]. In addition LRH-1 has a role in gastric, colon, pancreatic and breast cancers [15], [16], [17], [18], [19].

LRH-1 contributes to breast cancer development and progression through its ability to induce aromatase expression in cancer associated stromal fibroblasts (CAFs) [17], [18], [20], [21]. In postmenopausal breast cancers, aromatase in adipose is the major source of mitogenic estrogen for growth of ER-positive breast tumors [22]. Aromatase activity is regulated primarily by transcriptional changes of its gene *CYP19A1*, via various tissue-specific promoters. Malignant breast epithelial cells secrete prostaglandin E_2_ (PGE_2_), allowing increased LRH-1 expression and LRH-1 mediated binding and transcriptional activation of aromatase promoter PI.3/PII [18], [20], [21], [23]. The LRH-1 induced increase in local estrogen levels has a paracrine effect on neighbouring tumor cells causing an elevation in LRH-1 expression via the direct binding of ERα to its promoter [15]. LRH-1 can also regulate ERα expression in breast cancer cell lines [24] providing evidence of a positive feedback loop between LRH-1 and ERα within tumor epithelial cells.

Although LRH-1 is not basally expressed in normal mammary tissue, high expression has been demonstrated in the epithelial compartment of both invasive ductal carcinoma and ductal carcinoma *in situ* [15], [17], [18]. LRH-1 expression in human tumors correlated with that of other genes involved in steroid synthesis, including P450 side-chain cleavage, 3β-hydroxysteroid dehydrogenase and the Steroidogenic Acute Regulatory protein, suggesting that LRH-1 may influence *in situ* steroidogenesis in breast cancer [17].

LRH-1 mediates the mitogenic effect of estrogen in breast cancer cells since siRNA-mediated knockdown of LRH-1 inhibits estrogen-induced MCF-7 cell proliferation [15]. Recently, we have demonstrated that LRH-1 not only enables the migration and invasion of breast cancer cell lines but also increases the tumorigenic potential of the normal mammary epithelial cell line MCF-10A [25].

To identify cellular pathways regulated by LRH-1 in breast cancer epithelial cells, we performed microarrays to identify genes which were transcriptionally regulated by LRH-1 in MCF-7 cells which had LRH-1 over-expressed or knocked down (data not shown). Interestingly one of the most significantly altered genes, caused by modulation of LRH-1 expression, was Growth Regulation by Estrogen in Breast Cancer (GREB1). Therefore we aimed to elucidate mechanisms via which LRH-1 regulated GREB1 transcription.

In the present study we demonstrate co-localisation of LRH-1 and ERα on three critical ERα response elements (EREs) on the GREB1 promoter to activate transcription and cell proliferation. LRH-1 bound directly to ERE sequences present on the promoters of two well characterised, estrogen responsive genes GREB1 and Trefoil Factor 1 (TFF2 or pS2). These findings indicate that LRH-1 acts synergistically with ERα to induce transcription of GREB1 and unravels a new mechanism of action for LRH-1 in inducing cancer cell proliferation.

## Results

### Effects of LRH-1 on GREB1 expression in MCF-7 cells

**Figure 1:**
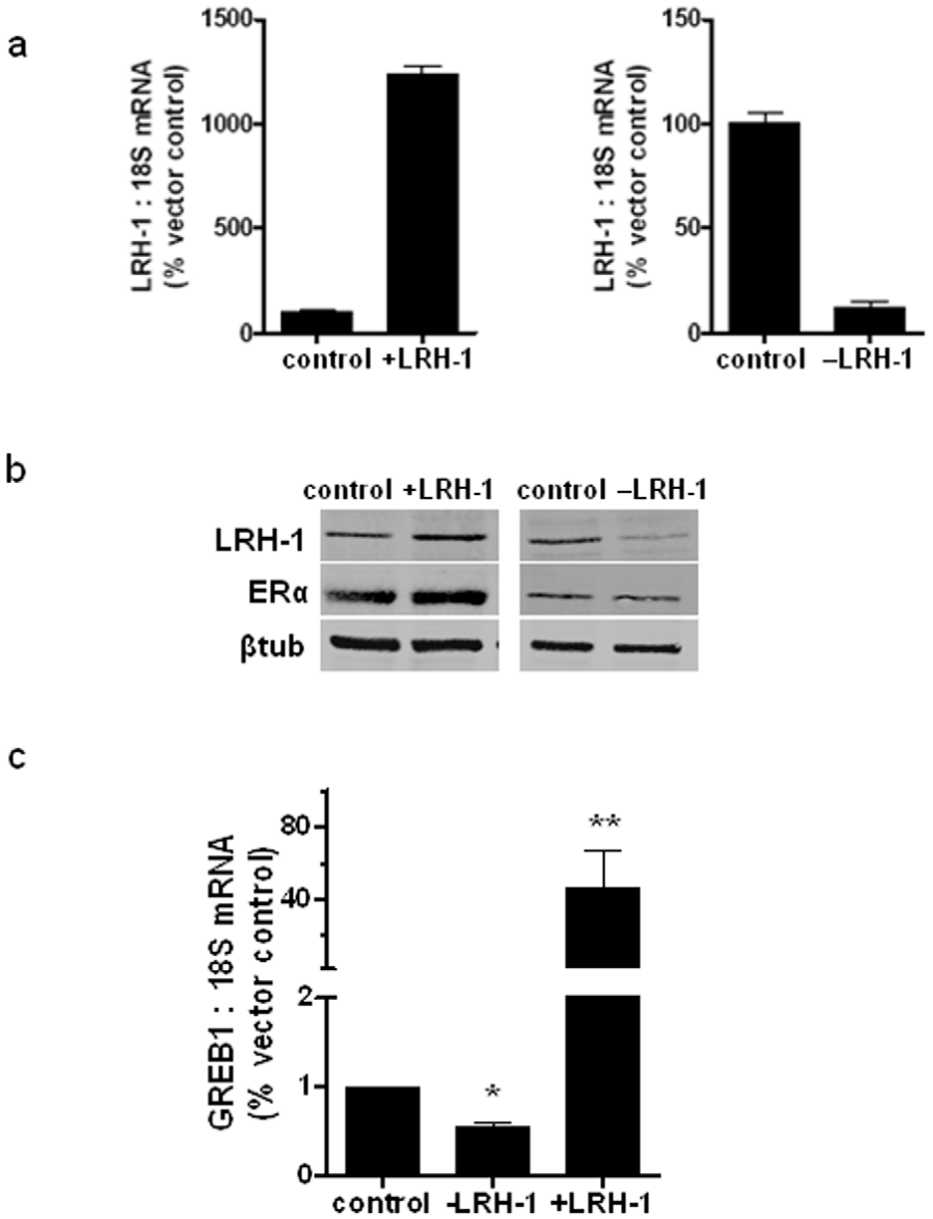
Modulation of LRH-1 expression in transcriptionally regulates GREB1. (a) Changes in LRH-1 mRNA and (b) protein levels in MCF-7 cells transfected with siRNA for LRH-1 (−LRH-1) or control; with pcDNA only or LRH-1-pcDNA (+LRH-1) constructs 24 h post transfection. (c) The expression levels of GREB1 in response to LRH-1 knockdown (siRNA) and over-expression (+LRH-1). Data were presented as % fold change compared to controls of the normalized expression levels, as mean ± SD, n = 3 separate experiments.

MCF-7 cells were transfected with either an LRH-1 – specific shRNA, a control shRNA, an expression vector encoding full-length human LRH-1 cDNA or an empty expression vector. Transfection with LRH-1 expression vector increased LRH-1 mRNA (12-fold) (Figure 1A) and protein expression (Figure 1B) compared to vector only transfected cells. Transfection with an expression plasmid encoding an LRH-1 – specific shRNA reduced endogenous LRH-1 mRNA levels in MCF-7 cells by approximately 5-fold compared to control shRNA – transfected cells 24 h post transfection (Figure 1A). This was reflected in a reduction in LRH- 1 protein by western blot analysis (Figure 1B). Having confirmed over- and under- expression of LRH-1 in MCF-7 cells, we next measured expression of GREB1, a gene previously identified in microarray data sets as significantly regulated by LRH-1 (data not shown). In LRH-1 over-expressing cells, GREB1 expression was elevated 40-fold while in LRH-1 knock down cells, a 2-fold decrease in GREB1 expression was observed (Figure 1C). These results correlated with the microarray findings.

GREB1 is a well characterised ERα target gene [26], [27]. To determine whether the increase in GREB1 expression could be attributed to LRH-1 mediated changes in ERα expression, we assessed protein expression by western blot. There was an increase observed in ERα levels in LRH-1 over-expressing cells, however the knockdown did not demonstrate a notable decrease (Figure 1b).

**Figure 2:**
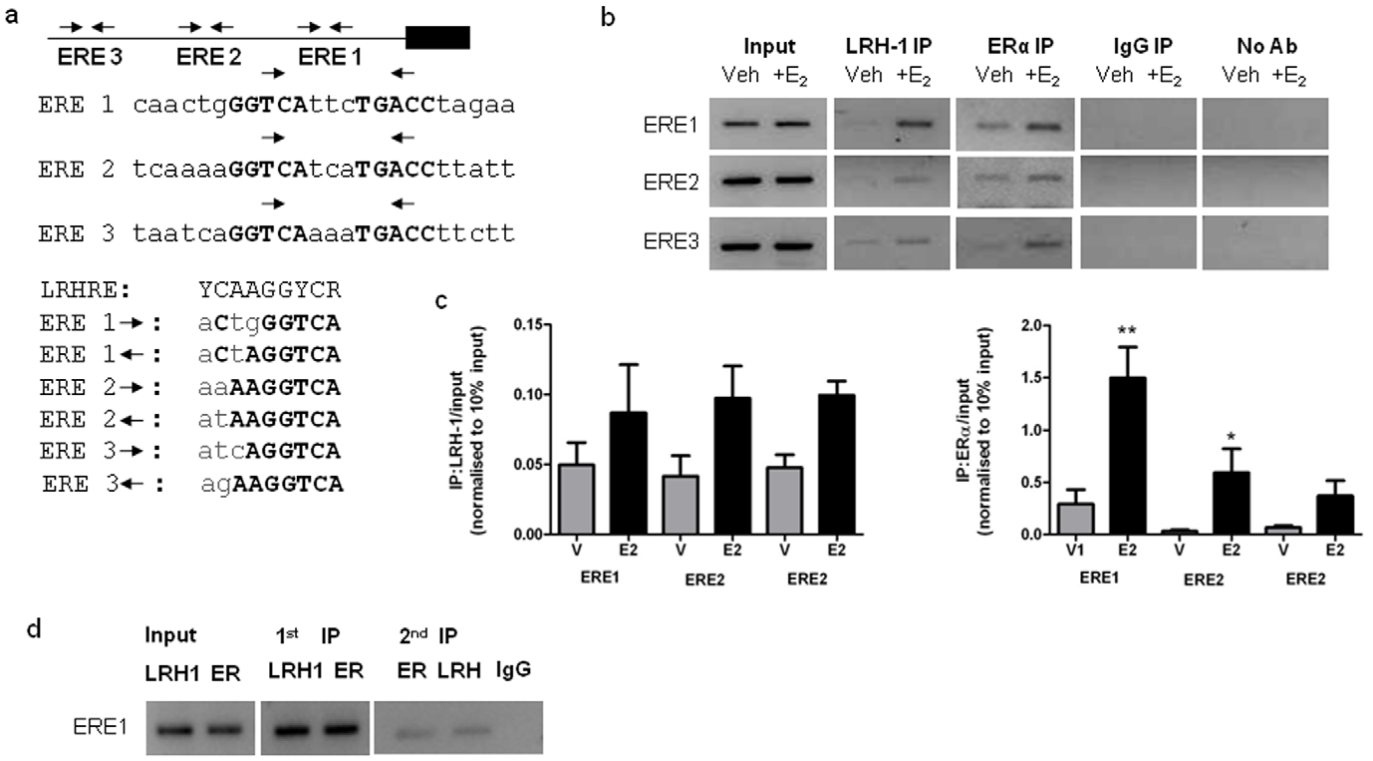
LRH-1 binds to three ERE sites within the GREB1 promoter. (a) Location of regulatory EREs on the distal and proximal GREB1 promoter, highlighting (bold) sequence similarity of the LRH-1 nuclear receptor half site within the ERE palindrome. (b) Chromatin immunoprecipitation (ChIP) showing occupancy of LRH-1 on the three EREs where ERα binds in the presence or absence of estradiol. Immunoprecipitation was performed with anti-LRH-1 and ERα antibodies on chromatin isolated from MCF-7 cells treated with vehicle or 10 nM 17β-estradiol for 45 mins. (c) The precipitated chromatin was analyzed by quantitative real-time PCR to demonstrate relative occupancy using the delta delta C_t_ method. Data is normalised to 10% of input. Data is represented from 3 or more separate treatments and separate ChIP experiments. (d) Sequential ChIP demonstrating co-localisation of ERα and LRH-1 on ERE1 of the *GREB1* promoter. Figures are representative of 3 or more separate ChIP experiments.

Estrogen regulation of GREB1 transcription is mediated by 3 estrogen response elements (EREs) located in the distal and proximal promoter regions [27] (Figure 2A). Analysis of the GREB1 promoter sequence for LRH-1 nuclear receptor half sites (LRHRE) containing the YCAAGGYCR motif (where Y is a pyrimidine and R is a purine) identified three potential LRHRE sites (Figure 2A). Interestingly these putative LRHREs were located within the ERα nuclear receptor recognition sites (ERE) found in the distal and proximal GREB1 promoter [27], [28]. DNA binding sites for ERα and LRH-1 demonstrated significant sequence similarity (highlighted in Figure 2A), raising the possibility that LRH-1 binding could occur within the palindromic ERE sequence (PuGGTCAnnnTGACCPy) as depicted in Figure 2A. This led to the hypothesis that LRH-1 could bind directly to the ERE of these LRH-1/ERα target genes. To address this hypothesis we examined the promoter regulation of GREB1 in more detail.

### LRH-1 is recruited with ERα on the GREB1 promoter

To demonstrate the interaction between LRH-1 and the EREs at the GREB1 promoter, we used chromatin immunoprecipitation (ChIP). We observed interaction of endogenous ERα and LRH-1 on these three ERE sites (Figure 2B) suggesting a direct role of LRH-1 in transcriptional activation of GREB1. Following estradiol treatment for 45 minutes, binding of both ERα and LRH-1 to the three ERE motifs was increased (Figure 2B and 2C) suggesting increased expression of LRH-1 and/or an estradiol-enhanced recruitment with ERα to the promoter.

To assess whether ERα and LRH-1 simultaneously occupy the GREB1 ERE, we used sequential chromatin immunoprecipitation (SeqChIP) on chromatin obtained from MCF-7 cells untreated with estradiol. The first SeqChIP experiment involved ChIP with the LRH-1 antibody first, followed by a second ChIP with the ERα antibody. PCR of the resulting DNA with primers specific to ERE1 indicated co-occupancy of LRH-1 and ERα on the proximal ERE under basal conditions (Figure 2D). As validation, a second SeqChIP where ERα antibody was used in the first ChIP, followed by LRH-1 antibody for a second ChIP, to demonstrate the same result. Band intensity for the first ChIP (for both LRH-1 and ERα antibodies) was greater, when compared to results in Figure 2B, as the amount of chromatin introduced to the reaction was altered. Collectively, these results demonstrate the occupancy of both ERα and LRH-1 at the EREs located in the GREB1 promoter region, and suggest a direct relationship between LRH-1 and ERα in the regulation of GREB1 gene expression.

### LRH-1 binds directly to EREs on the GREB1 and pS2 promoters

**Figure 3:**
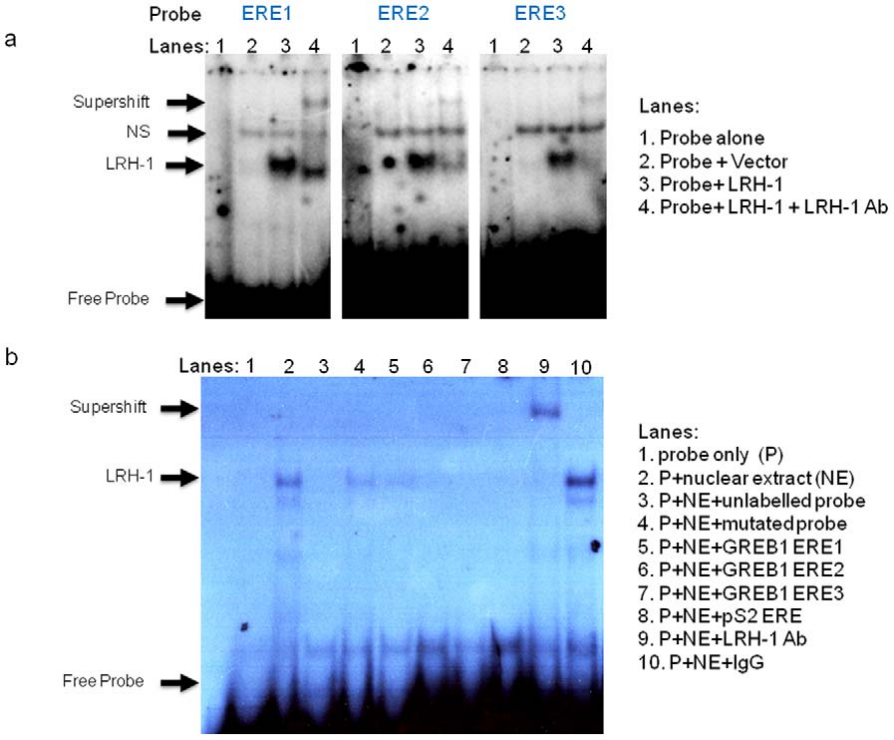
LRH-1 binds to specific ERE sequences of the GREB1 and pS2 promoters. (a) EMSA showing binding of LRH-1 to the EREs present in the GREB1 promoter. Radiolabeled ERE1-GREB1, ERE2-GREB1 and ERE3-GREB1 probes were incubated with *in vitro* translated LRH-1 protein. *In vitro* translation of the empty vector was used as a negative control. Anti-LRH-1 antibody was added in addition to the probe and the LRH-1 protein to indicate specificity of protein binding. (b) EMSA showing binding of LRH-1 to the EREs present in the GREB1 and pS2 promoters. Radiolabeled LRHRE probe (containing the LRH-1 response element derived from the aromatase promoter), whole cell nuclear extracts infected with a LRH-1 viral construct were incubated with various oligonucleotides (as listed in the figure) including unlabeled LRHRE, mutated LRHRE, ERE1-GREB1, ERE2-GREB1, ERE3-GREB1 and ERE-pS2 which were added in 200 fold excess. Anti-LRH-1 antibody and IgG were also added in addition to the probe and the nuclear extract to indicate specificity of protein binding.

As ChIP localises transcription factor binding only to the general vicinity of target sequences within genomic DNA (200–300 bp), we next confirmed that LRH-1 can bind directly to these EREs using competition electrophoretic mobility shift assays (EMSA, Figure 3A and 3B).

LRH-1 bound to all 3 GREB1 ERE probes and this LRH-1-DNA complex was supershifted with addition of an LRH-1 antibody (Figure 3A). Furthermore displacement of binding of GREB1 ERE probes to a probe containing a LRH-1 response element (LRHRE) (from the well validated LRH-1 target gene CYP19A1 PII promoter) was measured. Nuclear extracts form a complex with the LRHRE probe from PII promoter and this complex was supershifted in the presence of anti-LRH-1 antibody but not the control immunoglobulin G (Figure 3B lanes 9 and 10). Partial displacement of the LRH-1/LRHRE complex by ERE1 indicated a weak interaction (Figure 3, lane 5), while complete displacement by ERE2 and ERE3 demonstrated strong binding to these sequences (Figure 3, lanes 6 and 7). Furthermore LRH-1 also interacted with the ERE sequence from the pS2 promoter (Figure 3, lane 8). These results provide evidence for the direct interaction of LRH-1 protein with the palindromic ERE motifs located on the promoter regions of two well characterised ERα target genes, GREB1 and pS2 (TFF1).

### Transcriptional activation of ERE-containing luciferase reporters by LRH-1 reflects a synergistic action with ERα

In order to determine if LRH-1 could increase transcription of ERE-containing reporter constructs, expression plasmids encoding either ERα or LRH-1 were transfected with a luciferase reporter driven by two copies of either a consensus palindromic ERE (2×ERE), or the GREB1 ERE2 (GREB-ERE2). These reporter constructs were chosen for their sequence variability. The consensus ERE lacks the 5′ nucleotides known to support LRH-1 binding; while the GREB-ERE2 sequence contains the LRHRE motif (Figure 2A). Hence the reporter with the consensus ERE would not be expected to respond to LRH-1.

**Figure 4:**
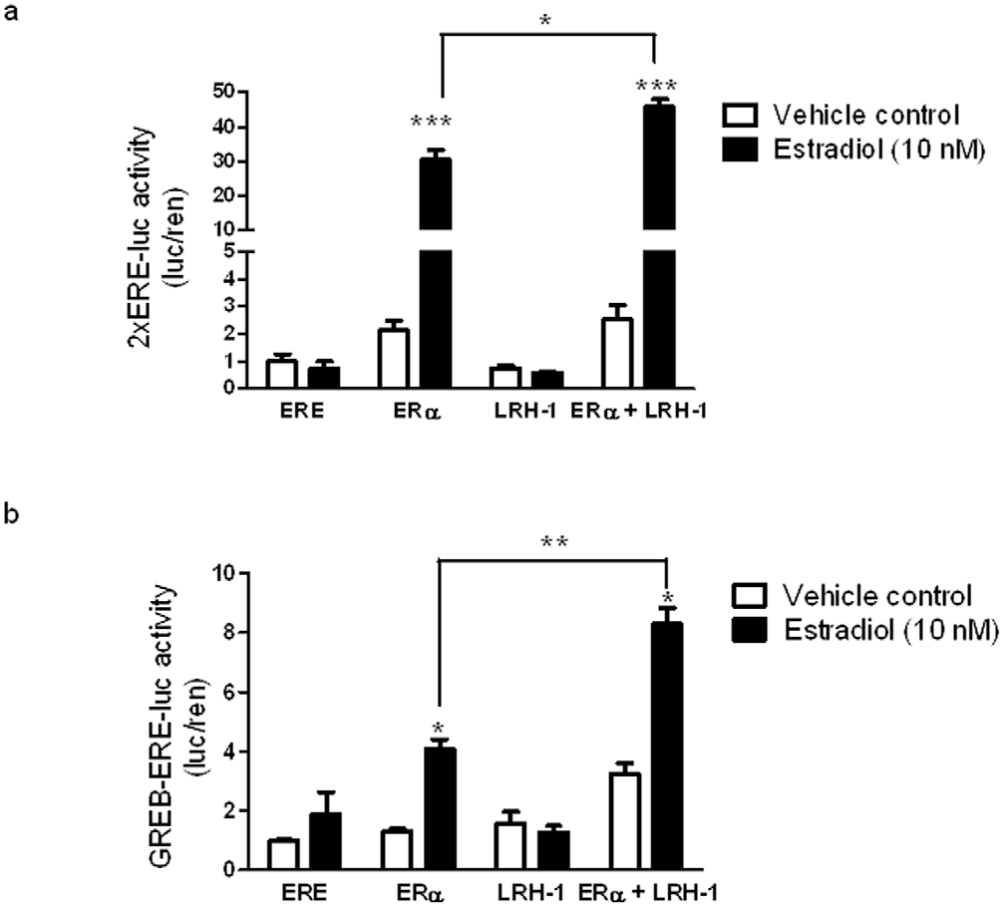
LRH-1 acts synergistically with ERα to activate ERE containing promoters. Transcriptional activation of (a) 2×ERE and (b) GREB-ERE2 luciferase reporters by ERα and LRH-1 with vehicle (veh) or 10 nM 17β-estradiol (E2). Estrogen-deprived MCF-7 cells were over expressed with LRH-1 or ERα alone, or in combination with the appropriate reporter construct. Cells were treated with 17β-estradiol for 16 h prior to luciferase assays. Data is presented as mean+SE, n = 3 separate experiments, treatments in triplicate per experiment. *P<0.05, *P<0.01, ***P<0.001 compared to vehicle control unless indicated by reference line.

Transfection with ERα increased activities of both reporters, and as expected, this induction was significantly enhanced by 10 nM estradiol treatment (Figures 4A and 4B). Transfection of LRH-1 alone (with or without estradiol treatment) did not show a difference in 2×ERE reporter activity (Figure 4A). However, LRH-1 alone caused a slight increase on GREB-ERE2 promoter activation but this was not altered by estradiol treatment (Figure 4B). The difference in sequence of the ERE palindrome between the two reporter constructs (with the GREB-ERE2 having a consensus LRHRE) could explain the small increment of LRH-1 induced reporter activity and sequence specific affinity of LRH-1 binding to EREs.

Cotransfection of ERα and LRH-1 caused a 2-fold increase in activity of both reporter constructs (Figures 4A and B). Interestingly with estradiol treatment there was a significant increase in reporter activity when compared promoter activity with ERα alone and estradiol treatment (Figures 4A and B). These results suggest that LRH-1 may enhance ligand dependent activity of ERα on transcriptional regulation of a subset of target genes.

### Effects of LRH-1 on GREB-1 expression and estradiol-dependent cell proliferation

**Figure 5:**
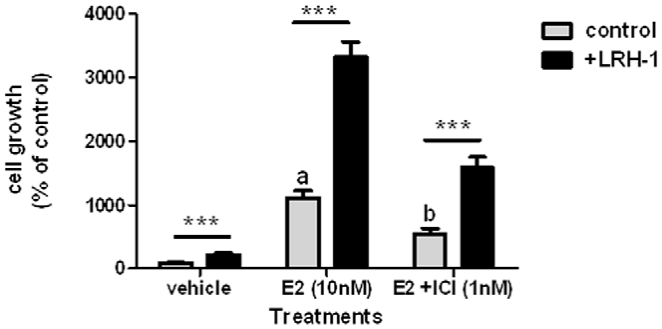
LRH-1 induces cell proliferation in 17β-estradiol and ICI 182,780 treated cells. Cell proliferation was measured in pcDNA alone transfected, estrogen-deprived MCF-7 cells (control) or LRH-1 over-expressing (+LRH-1) MCF-7 cells treated with vehicle, 10 nM 17β-estradiol (E2) or 10 nM 17β-estradiol and 1 nM ICI 182,780, an ERα antagonist for 5 days. Data is presented as mean+SEM, n = 3 separate experiments, triplicate treatments per experiment, ***P<0.001 compared to control transfected cells; a,b P<0.001 compared to vehicle control.

**Figure 6:**
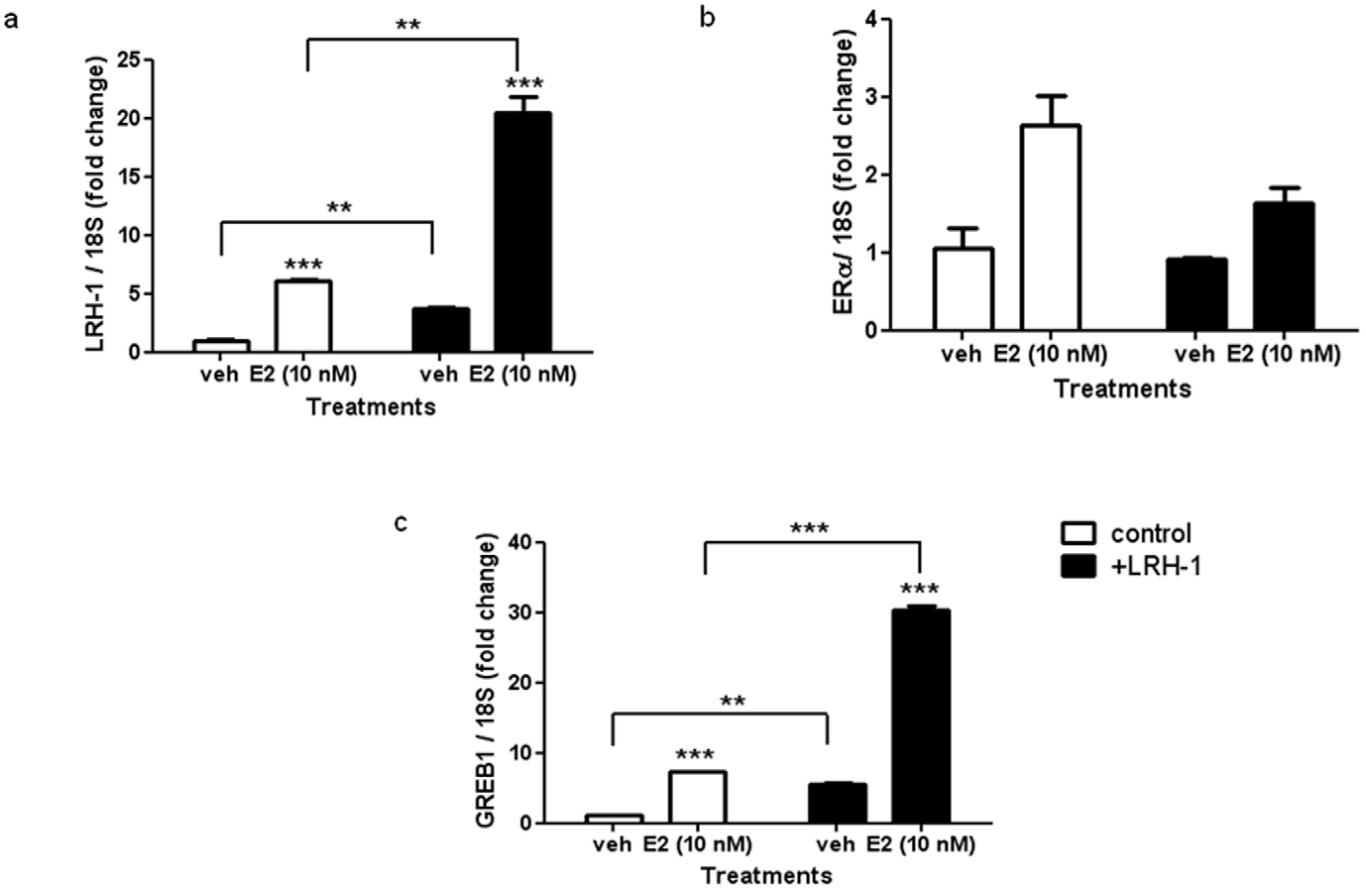
Synergistic effects of LRH-1 and 17β-estradiol treatment on GREB1 expression. Quantitation of (a) LRH-1, (b) GREB1 and (c) ERα mRNA expression in estrogen-deprived MCF-7 cells (control) or LRH-1 over-expressing (+LRH-1) MCF-7 cells treated with vehicle (veh) or 10 nM 17β-estradiol (E2) for 16 h. Data is presented as mean+SE, n = 3 separate experiments, triplicate treatments per experiment, **P<0.01, ***P<0.001 compared to vehicle control.

We next determined the effects of LRH-1 on estrogen-dependent cancer cell proliferation (Figure 5), and correlated cell proliferation to changes in transcript levels of LRH-1, ERα and GREB1 (Figure 6). LRH-1 over-expression in estrogen-depleted cells resulted in a significant 2-fold increase in cell proliferation (Figure 5). Estradiol treatment caused a 12-fold increase, while the additive effect of LRH-1 and estradiol treatment caused a 37-fold increase. In these cells, the combined increase LRH-1 and GREB1 expression positively correlates to proliferation.

Estradiol treatment caused a 6-fold increase in LRH-1 expression consistent with previous studies [15] (Figure 6A). In the stably transfected, serum depleted cells, a 4-fold LRH-1 over-expression compared to basal levels was observed. Interestingly, the addition of 17β-estradiol in LRH-1 over-expressing MCF-7 cells caused a 24-fold increase in LRH-1 compared to basal expression (Figure 6A). Similar expression patterns were observed for GREB1 under these treatment conditions (Figure 5C). Estradiol treatment induced a 4-fold increase in GREB1 expression, while a 32-fold increase was observed in estradiol treated, LRH-1 over-expressing cells (Figure 5C). These results show a clear positive correlation of LRH-1 and GREB1 transcript expression. ERα transcript levels remained relatively unchanged in response to the above treatment conditions. A two fold increase in ERα transcript was observed in response to estradiol treatment (Figure 5B). Over-expression of LRH-1 and estradiol treatment did not demonstrate a cumulative increment in ERα mRNA, as was observed for LRH-1 and GREB1 (Figure 5B). This lack of change in ERα expression is in concordance with previous reports [29]. Ligand activation of ERα and the increase in LRH-1 expression reflects the synergistic effects of LRH-1 and ERα observed on reporter transactivation assays (Figures 4A and 4B). These observations indicate the growth promoting role of LRH-1 and GREB-1 in an ERα dependent manner.

To examine the effect of LRH-1 on cell proliferation independent of estrogen signalling we also treated cells with a combination of estradiol and an ERα antagonist, ICI 182,780 (Figure 5). The presence of ICI 182,780 reduced estradiol-induced proliferation significantly 2-fold. In LRH-1 over-expressing cells treated with estradiol and ICI 182,780, a 2 fold decreased was also observed. However estradiol-mediated proliferation was significantly higher in LRH-1 over-expressing, ICI 182,780 treated cells compared to basal MCF7 cells (Figure 5). This implicates a role for LRH-1 in mediating a positive effect on tumour cell proliferation treated with ERα antagonists.

### LRH-1 regulation of GREB1 expression occurs independently of ERα expression

**Figure 7:**
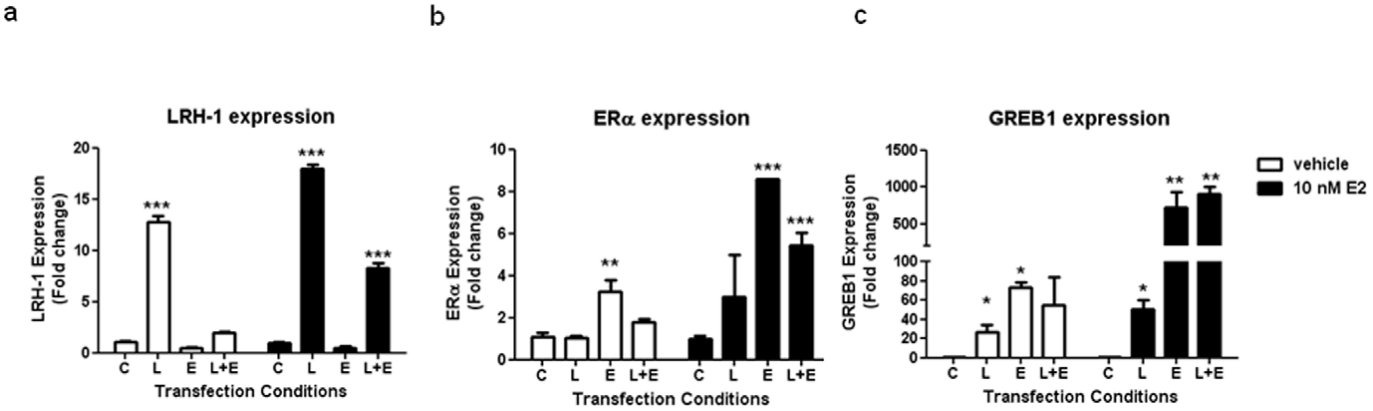
LRH-1 regulation of GREB1 expression in ER negative breast cancer cells. MDA-MB-231 cells were transfected with empty vector (C) or expression vectors for LRH-1 alone (L), ERα alone (E) or both LRH-1 and ERα (L+E). Cells were treated with vehicle or 10 nM 17β-estradiol (E2) for 16 h. Quantitation of (a) LRH-1, (b) ERα and (c) GREB1 mRNA expression. Data is presented as mean+SE, n = 3 separate experiments, ***P<0.001 compared to vehicle control.

In the ER-negative breast cancer cell line MDA-MB-231 cells, LRH-1 over-expression caused a significant, 26-fold increase in GREB-1 expression (Figure 7C). Co-transfection with LRH-1 and ERα with estradiol treatment demonstrated the synergistic effects on GREB1 expression as observed in MCF7 cells. This data demonstrates that LRH-1 is able to stimulate GREB1 expression independent of ERα signalling.

## Discussion

Little is known about the mechanisms of LRH-1 action in breast tumors. LRH-1 is abnormally expressed in 45% of all breast carcinomas and is positively correlated with tumor ER status [17]. Our finding suggests a novel association of LRH-1 and ERα in the regulation of GREB1 transcription and cell proliferation.

The gene encoding GREB1 was first identified as one of the key transcripts up-regulated in MCF-7 cells upon estradiol treatment and thus named after its function [30]. Its expression is positively correlated to ER-positive breast cancer in several clinical studies [26], [30, Rae, 2005 #406]. The role of GREB1 in cell proliferation was demonstrated with the suppression of GREB1 with siRNA, causing a significant reduction in cell proliferation [26]. Recently a monoclonal antibody for GREB1 was created, allowing the detection of a 216 kDa protein whose expression positively correlated to ERα expression in breast cancer cell lines and tumor samples, as well as to GREB1 mRNA transcript levels [31]. While expression of GREB1 protein appears predominantly nuclear, some cytoplasmic staining was also observed. GREB1 protein was expressed in tumor epithelial cells and CAFs [31]. As commercially available antibodies report variations in size of detected band in western blots and variability in cell localisation, the current study focussed on transcript expression analysis.

LRH-1 binds as a monomer to a specific sequence YCAAGGYCR. Analysis of DNA sequence motifs of ERE and LRH-1 nuclear receptor half site (LRHRE) indicated a sequence overlap suggesting that LRH-1 could bind to EREs of the GREB1 promoter. Our studies show direct binding of LRH-1 to the EREs of known ERα target genes GREB1 and pS2 (TFF1). We demonstrate specificity of LRH-1 binding to all 3 ERE sequence motifs with EMSAs. The recruitment of ERα and LRH-1 on distal and proximal GREB1 promoter EREs by ChIP was also demonstrated. By SeqChip experiments LRH-1 and ERα co-occupancy under basal conditions, to ERE1, the ERE most proximal to the start site was evident. ERα is thought to function as an underlying core transcriptional scaffold for interaction with other transcription factors such as the forkhead protein, FoxA1 [32]. Furthermore its actions are triggered by various cellular stimuli including growth factors such as Epidermal Growth Factor [32], [33]. This scaffolding function is observed in the regulation of GREB1 (located on chromosome 2) and pS2 (TFF1, located on chromosome 21), where estrogen stimulated ERα DNA binding resulted in the interaction or looping between the 2 chromosomal regions and significant enhancement of gene expression [34]. Our findings raise the possibility that LRH-1 may contribute to the variable actions of ERα mediated transcription in breast cancer cells.

In reporter transactivation assays, LRH-1 over-expression caused a modest increase in GREB-ERE reporter activity and no change on 2×ERE activity. This discrepancy in response reflects the importance of flanking sequences required for selectivity of LRH-1 binding to EREs. The two distal EREs located ∼20 kb upstream from the GREB1 transcriptional start site perform vital enhancer functions; ERα binding to these EREs allows chromatin looping and interaction with the proximal ERE to initiate GREB1 transcription [28]. Therefore the use of two copies of a single GREB1 ERE may not be sufficient to demonstrate the impact of LRH-1 and ERα on GREB1 transcription. However, the finding of most interest in the current study is the co-operative effect of ERα and LRH-1 expression in estradiol-treated conditions. This additive effect is observed consistently in mRNA expression analysis, promoter assays and ChIP experiments suggesting that LRH-1 may be associating with the ligand-activated ERα within a transcriptional complex at the EREs of GREB1 promoter.

LRH-1 is known to induce cancer cell proliferation [15], [16], [25], [35]. In breast cancer cells it enhances cell proliferation as a downstream effector of estradiol treatment [15]. In the present study, basal and LRH-1 over-expressing MCF-7 cells when treated with estradiol demonstrated a dramatic increase in GREB1 expression, and this correlated with increases in cell proliferation. The pattern of LRH-1 mRNA mirrored that of GREB1 while ERα mRNA levels were relatively unchanged under these treatment conditions. The expression data indicates that the activation of ERα, combined with the increase in the constitutively active LRH-1 expression, correlates to elevated GREB1 levels. These transcriptional changes are reflected in increased cell proliferation in LRH-1 over-expressing, estradiol-treated MCF-7 cells. In ER-positive breast cancers, GREB1 expression is correlated with high circulating estradiol levels [36]. In addition, LRH-1 expression is regulated by estradiol, reflected by the positive correlation of its expression in ER-positive tumors [15], [17].

In MCF-7 treated with the ERα inhibitor ICI 182,780, the presence of LRH-1 maintained increased proliferation indicating an ERα independent effect on cell proliferation previously not reported. This implicates that the presence of LRH-1 in tumours treated with selective ER modulators may account for estrogen –independent proliferation. Hence the regulation of GREB1 expression by LRH-1 identifies a novel mechanism for tumor cell proliferation.

Sun *et al* (2007) showed that the three EREs of the *GREB1* locus exhibit different degrees of ERα, coactivator, and polymerase II binding and suggested that different transcriptional regulators may be involved in modulating the ERα driven transcription from the 3 different GREB1 core promoters. Thus LRH-1 may be acting as a coregulator for some of these GREB1 core promoters, and it may possess different binding affinities to the EREs. We tested this hypothesis and demonstrated that LRH-1 was able to regulate ERα target genes such as GREB1 in ER-negative cancer cells such as MDA-MB-231 cells. LRH-1 also stimulated GREB1 expression significantly, however this effect was lower than that induced by ERα. The synergist actions of LRH-1 and ERα appeared to have the most potent effect on GREB1 expression and proliferation. It is a distinct possibility however that other ERα target genes may be transcriptionally regulated by LRH-1 in ER-negative tumor cells.

Another effect LRH-1 may confer is to maintain the expression of ERα target genes for longer durations post estradiol treatment. GREB1 expression rapidly induced within 2 h of estradiol treatment and maintained over a 48 h period [26], [30]. Could LRH-1 be a mechanism for the maintenance of ERα target gene expression? Assessing transcript levels of GREB1 in LRH-1 over-expressing and basal MCF-7 cells as varying time points post estradiol treatment would answer this question.

The synergistic increase in cell proliferation in LRH-1 over-expressing, estradiol treated cells could also reflect increased LRH-1 activity in addition to an increase in expression. While LRH-1 is constitutively active, it requires coactivators such as SRC1 and SRC3 to further activate its functions [37], [38]. As estrogen is needed to increase levels of SRC1 and SRC3 and has been shown to associate with the three GREB1 EREs [27], this is likely to cause activation of both ERα and LRH-1 at the transcriptional complexes as the proximal and distal promoters.

In summary, our findings provide a molecular mechanism for LRH-1 induced cell proliferation in ER-positive breast cancer cells; via the up-regulation of GREB1 expression. LRH-1 was able to induce GREB1 expression independent of ERα expression suggesting an estrogen independent effect on proliferation. This is the first evidence of LRH-1 binding to a subset of ERE palindrome motifs, as observed in GREB1 and pS2 gene promoters. In addition LRH-1 can colocalise with ERα (basally and in response to estradiol treatment) on proximal and distal promoter regions critical for the activation of GREB1 expression. As these proximal and distal regions connect via chromatin looping and may suggest a possible role for LRH-1 in aiding this transcriptional process. Whether LRH-1 partners with ERα as a heterodimer and whether LRH-1 can regulate ERα target genes in ER-negative tumor cells remains to be investigated.

Approximately 60% of premenopausal and 75% of postmenopausal breast cancer patients have ER-positive tumors. LRH-1 expression is positively correlated with tumor ERα status and here we demonstrate a co-operative effect of LRH-1 and ERα on GREB1 expression and cell proliferation. As some of the most successful therapies for breast cancer target the inhibition of ERα actions or of aromatase activity, the blockade of LRH-1 action in ER- positive tumors may provide further efficacy to current treatment regimes.

## Materials and Methods

### Plasmids

The human LRH-1 expression vector was generated as described previously [25]. The LRH-1 shRNA vector was constructed by cloning a double-stranded oligonucleotide targeting the appropriate sequence (GGATCCATCTTCCTGGTTA corresponding to nucleotides 1425-1407 of Genbank NM_205860) into pGeneclip hMGFP (Promega). The use of these constructs has been previously verified [25].

### Cell culture and transfection

All cells used were obtained from ATCC and grown in the recommended culture media and conditions. MCF-7 cells were cultured in DMEM (GIBCO) supplemented with 10% FBS, 50 U/ml penicillin and 50 µg/ml streptomycin at 37°C in 5% CO_2_ (all reagents obtained from Invitrogen). For LRH-1 knockdown, cells were transfected with either pGeneclip hMGFP-LRH-1 or pGeneclip hMGFP encoding a scrambled shRNA sequence as negative control (SABiosciences). For over-expression, cells were co-transfected with either pcDNA3.1+ or pcDNA3.1+LRH-1, and pGFP. Cells were transfected using the Nucleofector Kit V (Amaxa Biosystems), according to the manufacturer's instructions. GFP-positive cells were collected by fluorescence-activated cell sorting (FACS) 48 hours after transfection and replated or protein and RNA extracted as required. MDA-MB-231 cells were transfected with pcDNA containing constructs for LRH-1 and ERα using the Nucleofector Kit V as decribed previously (Chand *et al.*, 2010).

17β-estradiol (Sigma) treatments were performed in MCF-7-tet on cells that stably expressed the pTRE-LRH-1 construct. This allowed inducible treatment of LRH-1 with treatment with 1 ug/ml doxycyclin (Sigma). These cells were deprived of estradiol in phenol red free culture media supplemented with 5% charcoal stripped calf serum for 72 h prior to treatments with vehicle (ethanol) or 10 nM 17β-estradiol (Sigma) for 16 h. Doxycyclin treatment was performed for the same duration as vehicle or estradiol treatments to induce LRH-1 expression. Cells were then used for cell proliferation assays or RNA extracted for further studies.

### RNA extraction and quantitative real time PCR (qPCR)

Total RNA was extracted from cultured cells using the RNeasy kit (Qiagen), treated with DNaseI (Ambion), and quantified using a NanoDrop 1000 Spectrophotometer. First strand cDNA synthesis was accomplished using AMV reverse transcriptase (Promega) and random hexamers. For qPCR, cDNAs were diluted 1∶10 in water and amplified using SYBR Green chemistry on the LightCycler system (Roche), as previously described (Clyne *et al.* 2002). Primer sequences are outlined in Figure S1. Fold changes in expression of each gene were calculated using the delta delta C_t_ method [39] using 18S as the internal control.

### Western blot analysis

Protein extraction and western immunoblots for LRH-1 and β-tubulin were performed as described previously [25]. Protein bands were visualised using the Odyssey infrared imaging system and Odyssey 3.0 software (Licor Biosciences).

### Chromatin immunopreciptiation

MCF-7 cells were cultured for 3 days in phenol red free medium containing 5% CSS and then treated for 45 mins with vehicle (ethanol) or 10 nM 17-βestradiol (Sigma). Cells were fixed 10 min in PBS containing 1% formaldehyde, after which the reaction was stopped with addition of glycine. Chromatin was isolated and sheared according to protocols described previously [23]. Ten µl of sheared chromatin (300–500 bp) was collected as input and immunoprecipitation performed overnight with no antibody, 4 µg IgG or antibodies raised against human ERα (Santa Cruz) or LRH-1 (Abcam) separately or sequentially. Immunoprecipitated chromatin was eluted and reverse cross-linked according ChIP-IT Express (Active Motif) manufacturer's guide. Primers used for the amplification of ChIP chromatin are outlined in Figure S1. The precipitated chromatin was analyzed by quantitative real-time PCR to demonstrate relative occupancy using the delta delta C_t_ method. Data is normalised to 10% of input. Data is represented from 3 or more separate treatments and separate ChIP experiments.

### Electrophoretic mobility shift assays (EMSA)

Forward and reverse oligonucleotides (0.1 µg/µl) containing the consensus LRH-1 binding site (LRHRE) of aromatase promoter II (LRHRE sequences: Figure S1) were added in equal amounts and used as probes. Additional probes used included a mutated LRHRE sequence; GREB1 ERE1, GREB1 ERE2, GREB1 ERE3 and ERE of pS2 (see Figure S1 for all probe sequences). The concentrations of the forward and reverse oligonucleotides were 1 µg/µl. Probes were labelled and purified as described previously [40]. For competition experiments, 200-fold molar excess of mutant SFRE, GREB1 ERE, GREB1 ERE2, GREB1 ERE3 and ERE of pS2 annealed double-stranded oligonucleotides were included just before adding labelled probe to the reaction buffer (5× gel shift buffer, 0.5 mg/ml Poly dI.dC, 10 mg/ml BSA and 100 mM DTT). Protein was obtained eitherfrom whole cell extracts of sf9 insect cells infected with baculoviruses containing the cDNA of the human LRH-1; or *in vitro* translated empty expression vector (pcDNA3.1) or LRH-1 cDNA containing pcDNA vector construct. In vitro translation reactions were performed using thePromega TNT Quick Coupled Translation System (Roche). Mouse IgG (negative control) or anti-LRH-1 antibody (Abcam) were incubated on ice for 20 mins with whole cell extracts to demonstrate specificity of interactions. DNA-protein complexes were separated by electrophoresis on a 4% polyacrylamide gel in 0. 5× TBE buffer at 4°C.

### Luciferase reporter assays

HEK293 cells were plated into 48-well plates and transfected with 10 ng hLRH-1 pcDNA and or hERα pCDNA construct with 200 ng 2×ERE-luc (donated by Dr S Chu) or GREB-ERE2- constructs. The 2×ERE-luc reporter contains the palindromic ERE sequence: AGGTCACAGTGACCTgagctcAGGTCACAGTGACCT [41]. To construct the GREB-ERE reporter construct, complementary oligonucleotides encoding the GREB1 ERE2 (5′-3′: sense: TCTCAAAAGGTCATCATGACCTTATTGT, and antisense: ACAATAAGGTCATGATGACCTTTTGAGA) were annealed and ligated to form concatamers using T4 kinase and T4 ligase. DNA corresponding to 2 copies of the ERE sequence were purified, ligated into pGEMT-Easy, sequenced to confirm correct orientation, and then subcloned into the luciferase reporter vector pGL3basic. For transfections, DNA amounts were equalised by the addition of the empty vector (pcDNA3.1) construct and a 1∶3 DNA: Lipofectamine ratio used for the transfection as specified by manufacturers (Invitrogen). Cells were maintained in media supplemented with 5% charcoal stripped serum overnight after which cells were treated overnight with 10 nM 17 β-estradiol (Sigma).

### Cell proliferation assay

Transfected and treated cells, as detailed previously, were washed with PBS, subjected to trypsinisation. Cells were suspended in media supplemented with 10% FCS and Trypan Blue stain was added to the cell suspension. Viable cell number was quantitated using the Countess Automated Cell Counter according to the manufacturer's instructions (Invitrogen).

### Statistical analysis

All data are reported as mean ± SE for three or more experiments. Statistical analyses for experiments comparing two groups were performed by two-tailed Student's independent *t* test using GraphPad Prism 5.0 (GraphPad, La Jolla, CA, USA) and a *P* value of <0.05 was considered statistically significant.

## Supporting Information

Figure S1 **Sequences for qPCR Primers and EMSA probes.**
(DOC)
