# Supplementary material for: The Orphan Nuclear Receptor LRH-1 and ERα Activate GREB1 Expression to Induce Breast Cancer Cell Proliferation
Source: PLoS One. 2012 Feb 16;7(2):e31593. doi: 10.1371/journal.pone.0031593 (PMC3281101; doi:10.1371/journal.pone.0031593)
Supplement: Figure S1 — Sequences for qPCR Primers and EMSA probes. (DOC) [file pone.0031593.s001.doc]

| **Name** | **Orientation** | **Sequence 5’→3’** |
| --- | --- | --- |
| ***q-PCR Primers*** |  |  |
|  |  |  |
| LRH-1 | sense | ctgatactggaacttttgaa |
|  | antisense | cttcatttggtcatcaacctt |
|  |  |  |
| 18S | sense | cggctaccacatccaagga |
|  | antisense | gctggaattaccgcggct |
|  |  |  |
| GREB1 | sense | gtggtagccgagtggacaat |
|  | antisense | aaacccgtctgtggtacagc |
|  |  |  |
| ERα | sense | tgtccagccaccaaccagt |
|  | antisense | tttcaacattctccctcctctt |
|  |  |  |
| ***ChIP PCR Primers*** |  |  |
|  |  |  |
| GREB1 ERE1 | sense | gtggcaactgggtcattctga |
|  | antisense | cgacccacagaaatgaaaagg |
|  |  |  |
| GREB1 ERE2 | sense | gccacctctgcaggattgta |
|  | antisense | caaaacagagcaaggccaaa |
|  |  |  |
| GREB1 ERE3 | sense | tgtgctcagtgacccttgtg |
|  | antisense | Ctgccccaacaactgaaaga |
|  |  |  |
| ***EMSA Oligos*** |  |  |
|  |  |  |
| LRHRE | sense | gactctaccaaggtcagaaatgct |
|  | antisense | agcatttctgaccttggtagagtc |
|  |  |  |
| mutated LRHRE | sense | gactctacctttttcagaaatgct |
|  | antisense | agcatttctgaaaaaggtagagtc |
|  |  |  |
| *GREB1* ERE1 | sense | ggcaactgggtcattctgacctagaagc |
|  | antisense | gcttctaggtcagaatgacccagttgcc |
|  |  |  |
| *GREB1* ERE2 | sense | tctcaaaaggtcatcatgaccttattgt |
|  | antisense | acaataaggtcatgatgaccttttgaga |
|  |  |  |
| *GREB1* ERE3 | sense | gataatcaggtcaaaatgaccttctttc |
|  | antisense | gaaagaaggtcattttgacctgattatc |
|  |  |  |
| ERE of *pS2* | sense | cccctgcaaggtcacggtggccaccccg |
|  | antisense | cggggtggccaccgtgaccttgcagggg |
